# Posterior Reversible Encephalopathy Syndrome: A Clinico-Radiological Approach

**DOI:** 10.7759/cureus.72431

**Published:** 2024-10-26

**Authors:** Bhargavi Paladi, Jyotsna Yarlagadda, Tarani Chetana Naga Sai, M.L. Neeharika

**Affiliations:** 1 Department of Radiology and Imageology, Nizam's Institute of Medical Sciences, Hyderabad, IND; 2 Department of Neurology, Nizam's Institute of Medical Sciences, Hyderabad, IND

**Keywords:** clinico-radiological, haemorrhagic pres, parieto-occipital, posterior reversible encephalopathy syndrome (pres), pres with spinal cord involvement

## Abstract

Introduction

Posterior reversible encephalopathy syndrome (PRES) is a self-limiting neurological condition usually seen in young to middle-aged adults. Although most cases occur in the context of hypertension, PRES is not uncommon in normotensives. PRES is a clinico-radiological diagnosis that embodies specific clinical features, risk factors, and imaging findings. This study analyzes the clinico-radiological profile, typical and atypical features, and outcomes of patients presenting with PRES.

Methodology

This prospective observational study spanned 18 months and included 38 patients with suspected PRES who were referred from the neurology department. The patients’ brain MRIs were evaluated, and the affected regions and their signal intensity depicted in the T1-weighted, T2-weighted, fluid attenuation inversion recovery (FLAIR), and diffusion-weighted images were recorded. The pattern of involvement, atypical findings, and predisposing factors were examined.

Results

The subcortical white matter of the parieto-occipital lobes was the most typical area affected. The atypical regions affected were the cerebellum, thalamus, brainstem, and basal ganglia. Three cases exhibited isolated involvement of infratentorial structures. Spinal cord involvement was observed in two cases, of which one demonstrated dorsal cord involvement.

Conclusion

Most lesions are reversible. Long-term follow-up is recommended in hemorrhagic PRES and PRES with spinal cord involvement. Radiologists should be aware of the risk factors, and atypical clinical and imaging features to enable an early diagnosis and prevent further complications.

## Introduction

Posterior reversible encephalopathy syndrome (PRES) is a self-limiting neurological condition characterized by vasogenic edema predominantly in parieto-occipital lobes caused by sudden changes in blood pressure due to the susceptibility of posterior cerebral circulation [1]. This susceptibility is assumed to be a result of sparse sympathetic innervation [2]. PRES is a clinico-radiological diagnosis, with specific clinical features, risk factors, and characteristic imaging findings. In certain cases, however, it can occur in normotensive people and can have atypical imaging features.

PRES can occur at any age, but it is observed to affect young to middle-aged adults more frequently. Clinical features are of a broad spectrum and commonly include headache, seizures, visual disturbances, altered mental status, nausea, vomiting, and rarely focal neurological deficits. Precipitating factors include hypertension, eclampsia, autoimmune disorders, chemotherapy, immunosuppressant therapy, and sepsis.

PRES shows vasogenic edema in bilateral parieto-occipital lobes seen as cortical and subcortical hyperintensities on T2-weighted/fluid-attenuated inversion recovery (FLAIR) magnetic resonance imaging (MRI), with corresponding hypoattenuation on brain computed tomography (CT) [1,3]. Edema is clearly visualized on T2-weighted and FLAIR MRI sequences with better anatomical characterization than CT. Atypical areas of involvement include the brainstem, basal ganglia, corpus callosum, thalami, cerebellum, and other cerebral regions [4,5]. Involvement of frontal and temporal lobes is common. The central variant of PRES demonstrates brain stem or basal ganglia involvement with variable extension into thalami or periventricular white matter while sparing cortex and subcortical white matter [6]. Three patterns of anatomical distribution of vasogenic edema have been described: dominant parieto-occipital pattern, holo-hemispheric watershed pattern, and superior frontal sulcus pattern [4]. Mixed patterns of involvement are more common than isolated parieto-occipital involvement. Atypical features include diffusion restriction, hemorrhage, and post-contrast enhancement [3,4]. These unusual imaging features should be considered when dealing with complex cases.

On imaging, PRES must be differentiated from other conditions such as progressive multifocal leukoencephalopathy (PML), toxic leukoencephalopathy, and acute demyelinating encephalomyelitis. The presence of underlying immunosuppression, irreversibility, and peripheral diffusion restriction at the leading edge of the lesions favors the diagnosis of PML [7]. A history of toxic substance intake such as immunosuppressants or chemotherapy and diffusion restriction within white matter lesions is seen in toxic leukoencephalopathy [8]. In the presence of a typical history of prior vaccination or viral infection, white matter lesions that may show peripheral post-gadolinium enhancement, diffusion restriction, and CSF pleocytosis favor the diagnosis of acute demyelinating encephalomyelitis [9].

The diagnosis of PRES is supported by acute onset neurological symptoms, specific precipitating factors, characteristic imaging features, clinical improvement, and reversibility of lesions in the imaging on follow-up. In cases with atypical imaging findings and symptoms overlapping with other conditions, a detailed history of underlying precipitating factors and further evaluation using diffusion-weighted imaging (DWI), contrast-enhanced MRI, and follow-up imaging help to exclude other potential differentials. Follow-up imaging can be performed within one week to 10 days to ensure resolution. Due to significant clinical improvement, however, repeat imaging is seldom needed.

The aims of this study are to determine the typical and atypical regions of involvement and unusual imaging manifestations of PRES using MRI and to analyze the demographic, etiological, and clinico-radiological profiles and outcomes of patients presenting with PRES.

## Materials and methods

This prospective observational study spanned a period of 18 months from 2020 to 2022. It was conducted in the Department of Radiology, Nizam's Institute of Medical Sciences, Hyderabad, India. The study included 38 patients who presented with acute onset neurological symptoms in the setting of specific risk factors showing T2-weighted/FLAIR cortical and subcortical white matter hyperintensities on brain MRI, raising clinical suspicion of PRES. These patients were followed clinically and repeat imaging was performed in a few cases. Upon significant improvement on follow-up, they were diagnosed as PRES.

The study protocol involved acquiring three types of images: axial and coronal T2-weighted image with a repetition time of 5500 milliseconds (ms) and time to echo of 100 ms; axial T1-weighted image with a repetition time of 2000 ms and time to echo of 9 ms; and axial FLAIR image with a repetition time of 9000 ms, time to echo of 81 ms, and time of inversion of 2500 ms sequences with a 4 mm slice thickness. DWI with an apparent diffusion coefficient (ADC) map was also acquired. Brain CT and spine MRI performed in a few cases were reviewed. Repeat imaging was performed in five patients.

The presenting complaints, blood pressure recordings, and predisposing factors of the patients included in the study were analyzed. The regions involved and the signal intensity of the affected areas on T1-weighted, T2-weighted, FLAIR, and DWI images were recorded. Regions of involvement, atypical findings in the form of diffusion restriction, and hemorrhage on brain imaging were analyzed.

Descriptive statistics were elaborated in the form of mean/standard deviation for continuous variables and percentages for categorical variables. Categorical data were presented in a graphical manner wherever appropriate using pie charts.

## Results

**Figure 1:**
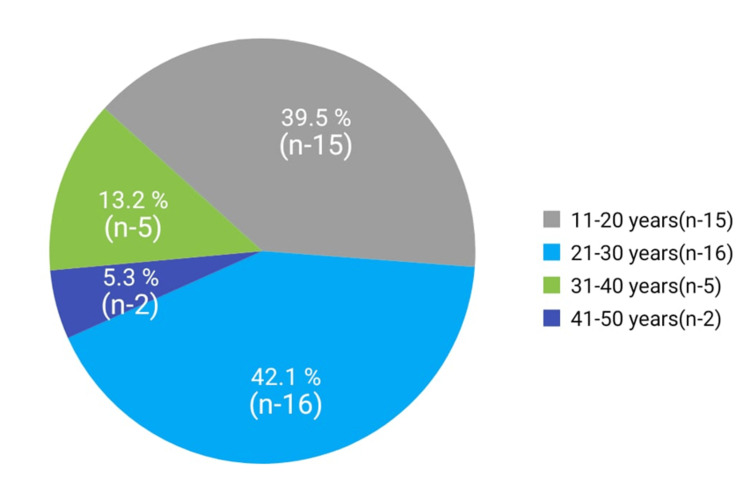
Pie chart showing the age distribution.

Of the 38 cases, 26 were female and 12 were male. The mean age was 24.3 ± 8.8 years. The age range was 13 to 50 years. Approximately 42% of the patients were aged 21 to 30 years (Figure 1).

Patients were presented with a range of symptoms. The most common presenting complaints in our study were recurrent episodes of generalized tonic-clonic seizures, followed by headache, nausea, and vomiting (Table 1).

**Table 1:** Symptomatology.

Presenting complaints	No. of patients (n)	Percentage of patients (%)
Seizures	32	84.2
Headache	20	52.6
Nausea & vomiting	7	18.4
Altered sensorium	5	13.1
Transient loss of vision/blurring of vision	4	10.4
Fever	3	7.8
Imbalance while walking	2	5.2
Difficulty in passing urine	1	2.6

The most common etiology/comorbidity was hypertension secondary to chronic renal disease (39.4%, n = 15), followed by pregnancy-induced hypertension (21%, n = 8; Table 2).

**Table 2:** Etiology or precipitating factors.

Precipitating factors	No. of patients (n)	Percentage of patients (%)
Hypertension secondary to chronic renal disease	15	39.4
Pregnancy-induced hypertension	8	21.0
Systemic lupus erythematosus with lupus nephritis	4	10.5
Hematological malignancies	3	7.8
Young-onset hypertension	2	5.2
Nephritic syndrome	2	5.2
Acute kidney injury	2	5.2
Immunosuppressants	1	2.6
Post blood transfusion	1	2.6

After receiving vincristine therapy for their acute lymphoblastic leukemia, two patients developed PRES. A case of post-renal transplantation on tacrolimus presented with COVID-19 pneumonia and esophageal candidiasis. After receiving fluconazole for esophageal infection, the patient developed PRES. This may be due to enzyme interactions, as fluconazole increases tacrolimus levels while decreasing its metabolism.

**Figure 2:**
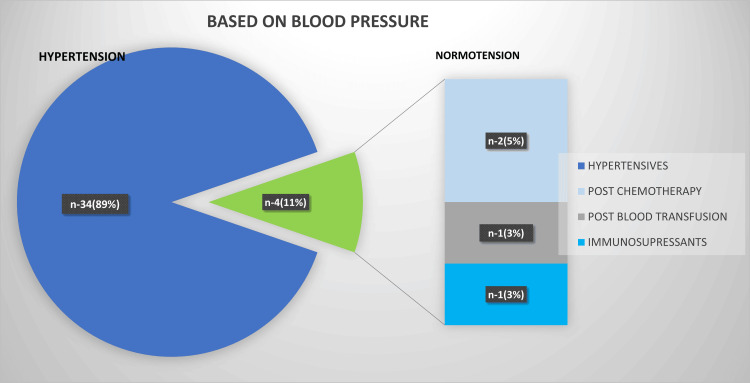
Pie diagram showing the distribution of cases on the basis of blood pressure.

Of the 38 cases, four normotensive patients developed PRES. Of these, two were post chemotherapy (vincristine), one was post immunosuppressant therapy (tacrolimus), and the other was post blood transfusion (Figure 2).

**Figure 3:**
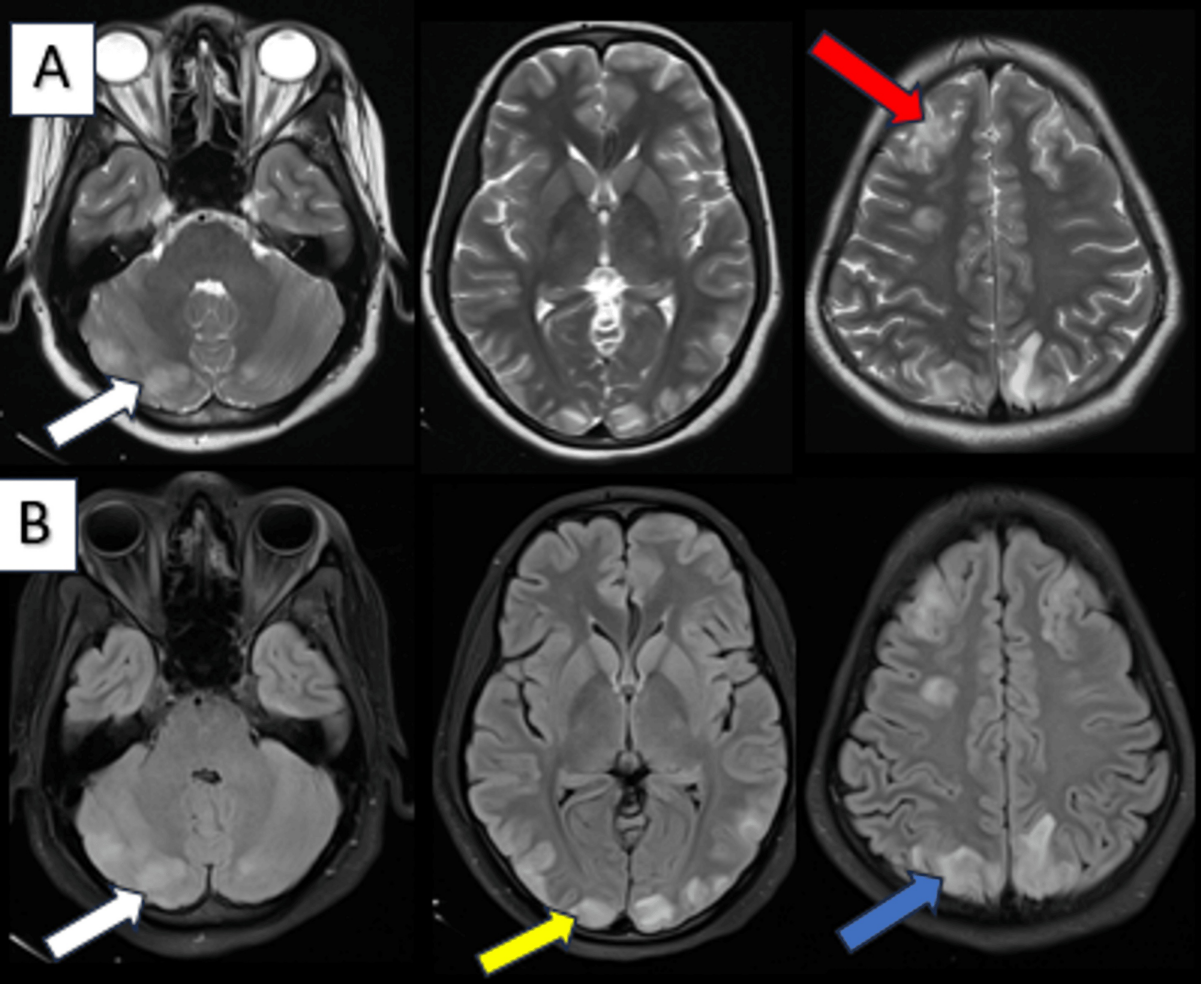
Axial T2W (A) and FLAIR (B) brain MRI. A 20-year-old female presented with seizures and altered sensorium diagnosed de novo as young onset hypertension. Axial MRI of the brain showed T2W/FLAIR hyperintensities in the subcortical white matter of the bilateral frontal (red arrow), parietal (blue arrow), and occipital (yellow arrow) lobes and the bilateral cerebellar hemispheres (white arrow). T2W: T2-weighted; FLAIR: fluid-attenuated inversion recovery.

**Figure 4:**
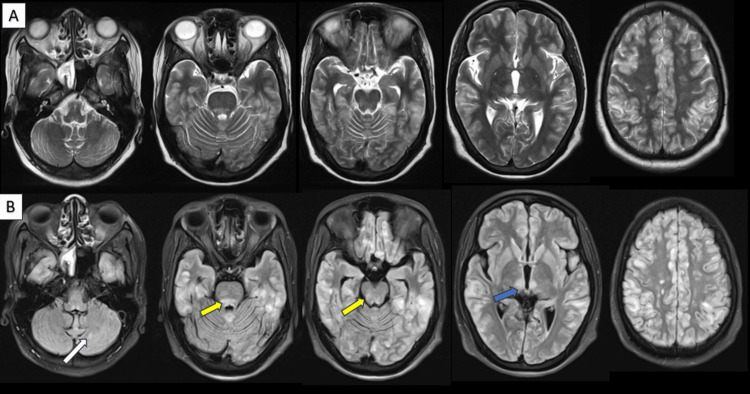
Axial T2W (A) and FLAIR (B) brain MRI. A 21-year-old female with chronic kidney disease presented with headaches and seizures. Axial brain MRI showed T2/FLAIR hyperintensities in the subcortical white matter of the left cerebellar hemisphere (white arrow), brain stem (yellow arrow), ​​​​​​​bilateral thalami (blue arrow), and bilateral cerebral hemispheres. T2W: T2-weighted; FLAIR: fluid-attenuated inversion recovery.

The most frequently involved typical locations were the subcortical white matter of the parietal lobes (92.1%, n = 35) and occipital lobes (100%, n = 33) (Figure 3). The most common atypical regions involved were the cerebellum in 60.5%, the thalamus in 15.7%, the brainstem in 13.1%, and the basal ganglia in 10.5% of cases (Figure 4). Bilateral symmetrical white matter hyperintensities on imaging accounted for 57.8% (n = 22) of the cases and bilateral asymmetrical white matter hyperintensities accounted for 42.1% (n = 16). Isolated involvement of infratentorial structures, namely, the cerebellum, brainstem, or spinal cord, was seen in three cases. Involvement of the spinal cord was seen in two cases, one of which specifically showed dorsal cord involvement and late resolution compared to the other.

**Figure 5:**
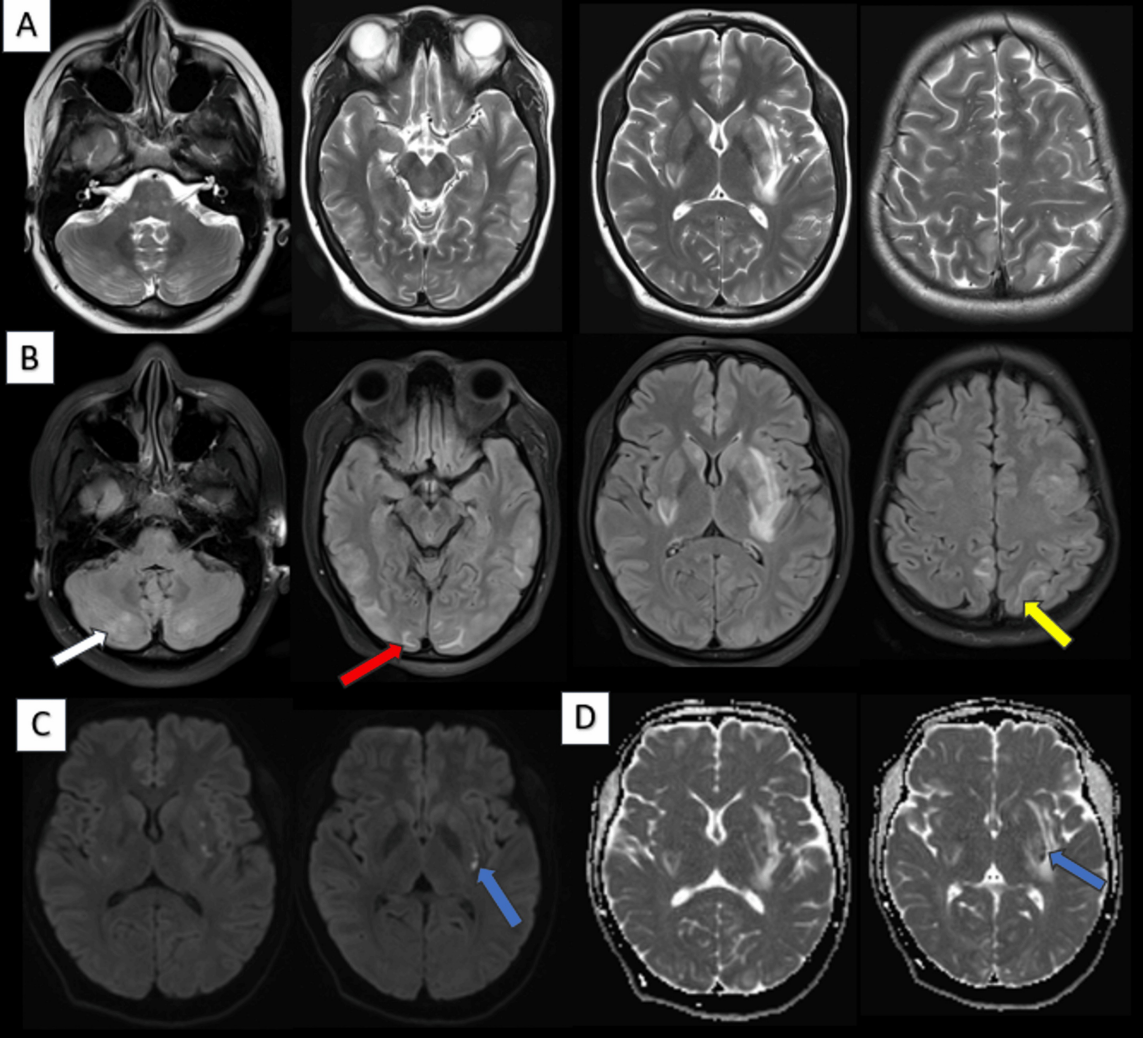
Axial T2W (A), FLAIR (B), DWI (C), and ADC (D) brain MRI. A 24-year-old female presented with a headache and two episodes of seizures on postpartum day seven. Axial brain MRI (A, B) showed T2W/FLAIR hyperintensities in both cerebellar hemispheres (white arrow), bilateral basal ganglia, subcortical white matter of the bilateral temporo-occipital (red arrow), parietal (yellow arrow), and left frontal lobes. Small foci of diffusion restriction with ADC reversal were observed in the bilateral basal ganglia (blue arrow, C and D). T2W: T2-weighted; FLAIR: fluid-attenuated inversion recovery; DWI: diffusion-weighted imaging; ADC: apparent diffusion coefficient.

Vasogenic edema primarily involves white matter with preserved grey-white matter differentiation and lacks diffusion restriction. DWI and ADC are valuable tools in differentiating PRES from acute ischemia, which is associated with cytotoxic edema and shows restricted diffusion. Though rare, areas of restricted diffusion seen in PRES superimposed on vasogenic edema. DWI should be a part of the imaging protocol in evaluating suspected cases of PRES. Diffusion restriction was noted in one case in our study (Figure 5) and the patient showed clinical improvement on follow-up.

**Figure 6:**
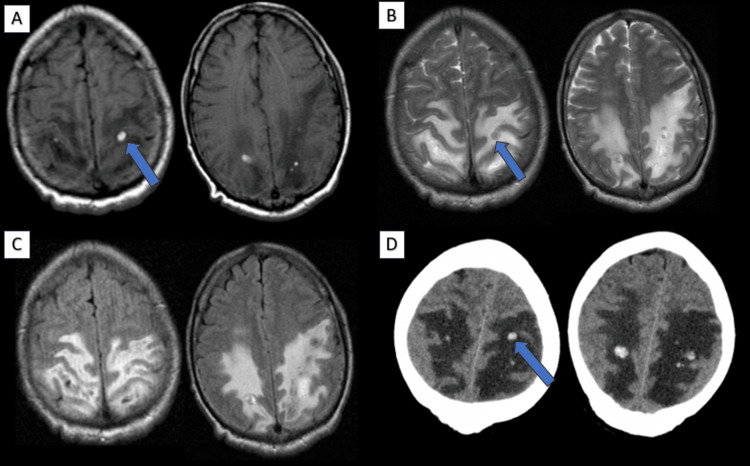
Axial T1W (A), T2W (B), and FLAIR (C) brain MRI and axial NECT of the brain (D) images. A 36-year-old male, a post-renal transplantation case on tacrolimus, presented with headache, seizures, and left upper and lower limb weakness. Brain MRI images showed hemorrhagic foci (blue arrow) with surrounding edema in the bilateral frontoparietal lobes. This patient had a residual focal neurological deficit in the form of left hemiplegia on follow-up. T1W: T1-weighted; T2W: T2-weighted; FLAIR: fluid-attenuated inversion recovery; NECT: non-enhanced computed tomography.

Hemorrhage in PRES is uncommon and may complicate the diagnosis. It can occur in various forms, such as focal parenchymal hematoma, petechial gyral hemorrhages, and subarachnoid hemorrhage. Susceptibility-weighted imaging is a valuable tool in detecting microhemorrhages. Hemorrhagic foci were seen in one case in our study in bilateral frontoparietal lobes (Figure 6). This patient had a residual focal neurological deficit in the form of right hemiplegia on follow-up.

**Figure 7:**
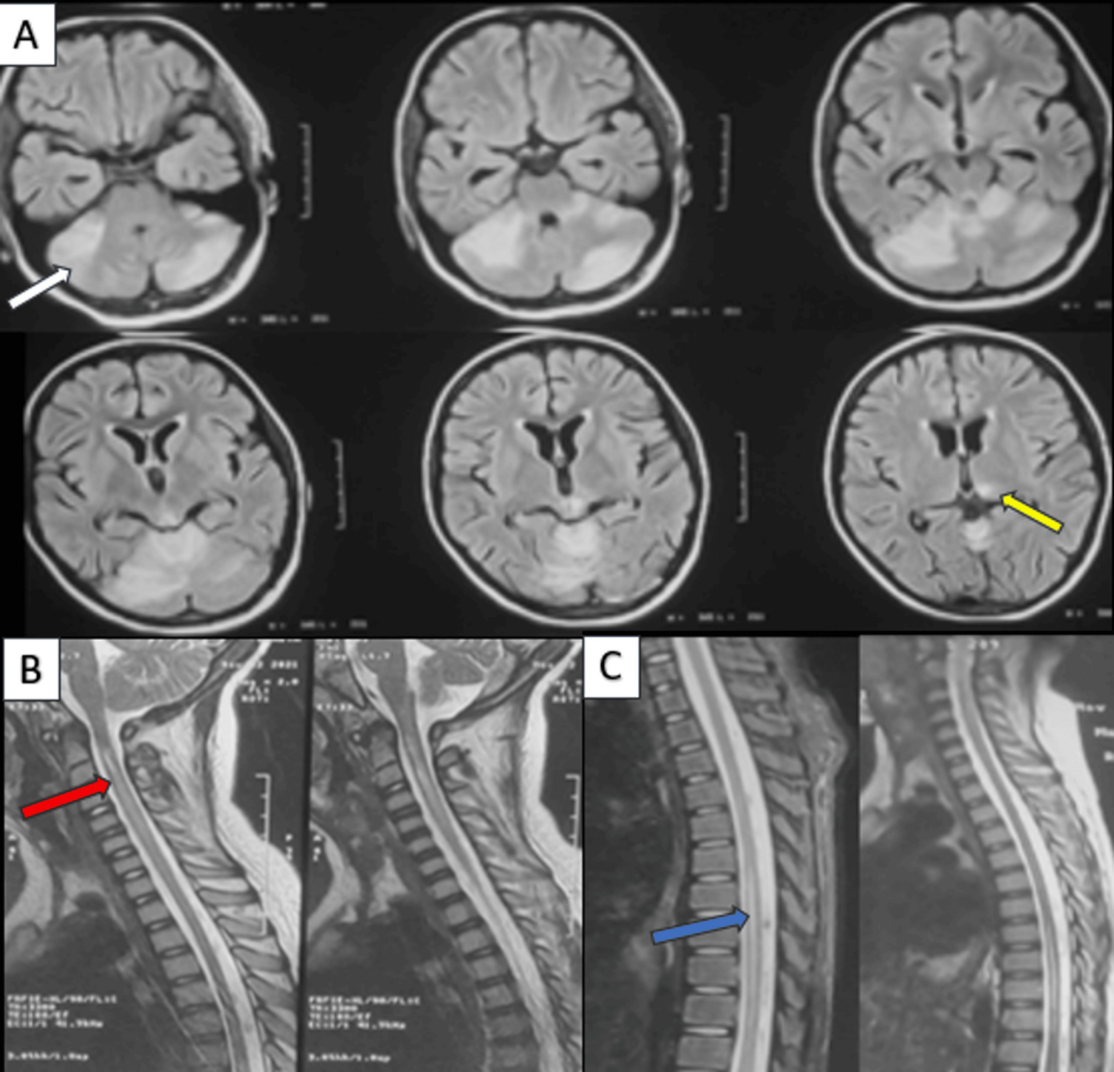
MRI axial FLAIR brain (A), sagittal T2W (B) cervical spine, and sagittal T2W (C) dorsal spine images. An 11-year-old female, a case of systemic lupus erythematosus, presented with headache, visual blurring, and swaying while walking. Her initial blood pressure recordings were high. Fundoscopy was suggestive of hypertensive retinopathy. She was treated with pulse steroids and antihypertensives and showed significant clinical improvement on day three. Brain MRI on day two of presentation revealed FLAIR (A) hyperintensities in both cerebellar hemispheres (white arrow) and left thalamus (yellow arrow). Cervical (B) and dorsal (C) spine MRI revealed central T2 hyperintensities in the cervical (red arrow) and thoracic (blue arrow) spinal cord. Follow-up brain imaging on day seven revealed complete resolution. Spine imaging after four weeks revealed significant resolution. T2W: T2-weighted; FLAIR: fluid-attenuated inversion recovery.

Two cases in our study showed spinal cord involvement, with cervical cord involvement in both cases. One of the cases showed involvement of both cerebellar hemispheres and cervical and dorsal cords (Figure 7). Dorsal cord involvement is very rare. Repeat imaging within one week showed complete resolution of cerebellar hyperintensities. Repeat imaging after one month showed significant resolution in cord changes but not complete resolution.

Patients were given supportive care. Case-specific treatment based on clinical features and risk factors was given. Treatment included antihypertensives and antiepileptics, and withholding the therapy in post-chemotherapy cases. Repeat imaging was done in five patients after six to eight days of the first MRI and it revealed complete resolution. One case of PRES with hemorrhagic foci showed a neurological deficit in the form of left hemiplegia. Though there was clinical improvement in the weakness, residual neurological deficit was present on follow-up after one year. Rest all cases on follow-up showed significant clinical improvement. Notable clinical recovery was seen in the spinal cord PRES case within three days and significant radiological resolution was evident after four weeks.

## Discussion

PRES is a self-limiting neurological condition usually seen in young to middle-aged adults. Although most of the cases occur alongside hypertension, its occurrence in normotensive people is not uncommon. Multiple theories have been proposed regarding the pathogenesis of PRES, the most widely accepted being the overwhelming of cerebral autoregulatory mechanisms due to acute changes in blood pressure causing dilatation of arterioles and leakage resulting in vasogenic edema [10]. In systemic inflammatory conditions, cytotoxins causing endothelial dysfunction and vasogenic edema is the most accepted hypothesis [11].

In a retrospective analysis of 92 patients, Raman et al. [12] found that 50% of the patients had peripartum/postpartum eclampsia (n = 46). In contrast, eclampsia (21%) and chronic kidney disease (39.4%, n = 15) accounted for the bulk of patients in our study, with no children under the age of 10. This could be due to the absence of a dedicated department for obstetrics and pediatrics in our hospital.

Atypical region involvement mostly occurred in the cerebellum and central zones (such as the basal ganglia, thalami, periventricular or deep white matter, brainstem, and spinal cord). We found that the occipital, parietal, and frontal lobes were still commonly involved. This suggests that PRES with atypical region involvement is often accompanied by typical region involvement. Diffusion restriction and hemorrhage are seen in very few cases (2.6%) in our study compared to other studies [5,12]. This could be due to a small sample size. Diffusion restriction seen in one case in our study showed clinical improvement on follow-up. Though restricted diffusion is present, PRES is reversible in most of the cases [13].

Kumai et al. [14] proposed that mild hypertension induces edema predominantly in the supratentorial white matter, whereas severe hypertension induces edema in infratentorial structures, the basal ganglia, and the thalamus. A similar finding we also noticed. In this study, cases of PRES with normotension commonly involved parieto-occipital lobes but not the basal ganglia or brainstem. This phenomenon could be due to high blood pressure, which may be required to produce changes in deep grey matter and the brainstem.

Few cases of isolated involvement of infratentorial structures sparing supratentorial structures have been reported [15,16]. There are three such cases in this study, two with isolated involvement of both cerebellar hemispheres and the other with involvement of the cerebellum and spinal cord. Isolated pons involvement has been reported [17]. This variant of PRES with the predominant involvement of the posterior fossa remains unexplained [18].

PRES with spinal cord involvement is a rare entity, first described by De Havenon et al. [19]. The pathophysiology is similar to that of typical PRES and is thought to be due to blood-spinal cord barrier disruption from the risk factors. The cervical cord seems to be more frequently affected than the dorsal cord. Since spine imaging is generally advised when neurological symptoms point to cord involvement, this variant is rarely documented, and dorsal cord involvement appears even rare. It is often difficult to differentiate such conditions from demyelinating disorders. In such cases, the time frame of clinical and radiological resolution, along with clinical history, helps to clinch the diagnosis.

One of the limitations of our study is the small sample size. Another is the nonavailability of dedicated obstetrics and pediatric departments in our center, which contributes to a lower percentage of PRES cases related to pregnancy-induced hypertension and pediatric cases in comparison to other studies.

## Conclusions

MRI features of PRES are vasogenic edema in the subcortical white matter of bilateral parieto-occipital lobes. Involvement of frontotemporal lobes and cerebellar hemispheres is also seen. It is not unusual for atypical regions such as the brain stem, thalami, basal ganglia, deep white matter, and splenium to be involved. Occasionally, the spinal cord may be affected as well.

PRES is a self-resolving condition in which lesions are reversible in the majority of cases. Long-term follow-up should be suggested in hemorrhagic PRES and PRES with spinal cord involvement. Radiologists should be aware of atypical clinical features, risk factors, and atypical imaging features to make early diagnosis and prevent further complications.
